# Salmonella Abortusequi-associated abortions in horses: Global epidemiology, diagnostic advances, prevention, antimicrobial resistance, and emerging control strategies

**DOI:** 10.14202/vetworld.2026.3498-3521

**Published:** 2026-08-11

**Authors:** Temirlan Bakishev, Zhassulan Baibolin, Sarsenbay Abdrakhmanov, Sergey Borovikov

**Affiliations:** 1 Institute of Animal Science and Veterinary, S. Seifullin Kazakh Agrotechnical Research University, 010011, Astana, Kazakhstan.

**Keywords:** antimicrobial resistance, bacteriophage therapy, diagnostic methods, equine abortion, epidemiology, One Health, Salmonella Abortusequi, whole-genome sequencing

## INTRODUCTION

*Salmonella enterica* subsp. *enterica* serovar Abortusequi is the principal etiological agent of salmonellosis-associated abortion in mares and is characterized by abortion, stillbirth, or the delivery of nonviable foals. Although horses are susceptible to *S. *Abortusequi infection, clinical disease is observed predominantly in pregnant mares, with abortion occurring most frequently during the later stages of gestation. Asymptomatic infections have also been reported in stallions, while neonatal foals may develop septicemic or enteric salmonellosis following exposure to infected mares. Aborted mares constitute the primary source of infection because they shed large quantities of bacteria through fetal membranes, amniotic fluid, vaginal discharges, and other reproductive secretions. In addition, clinically healthy carrier animals contribute to pathogen persistence within equine populations. Asymptomatic stallions may facilitate disease transmission through venereal contact and environmental contamination by shedding the organism in feces and urine, with bacterial excretion persisting for up to 60 days. Major transmission routes include contaminated feed, water, bedding, and horse-care equipment, whereas aborted fetuses, fetal membranes, and amniotic fluid serve as the principal reservoirs of infection. Both vertical and horizontal transmission have been documented. Following infection, *S. *Abortusequi colonizes the reproductive tract, inducing placentitis that compromises fetal viability. During acute infections, abortion may occur before fetal infection is established, whereas chronic infections permit bacterial invasion of the amniotic fluid and fetus from approximately the fourth month of gestation onward. Bacterial toxins and inflammatory mediators stimulate uterine contractions, resulting in fetal expulsion. Consequently, abortion often occurs during late pregnancy without preceding clinical signs, making early diagnosis particularly challenging [1–3]. Although *S. *Abortusequi is the predominant equine abortifacient *Salmonella* serovar, abortions caused by other *Salmonella* serovars have also been reported [4].

Equine abortion caused by *S. *Abortusequi results in substantial reproductive and economic losses through reduced broodmare fertility, diminished foal production, increased veterinary expenditures, and the implementation of disease control and biosecurity measures [5–7]. During outbreaks, the foal crop may decline by 20%–40%, depending on the severity of the epizootic situation [8]. Furthermore, abortions associated with mixed infections involving *Salmonella* and other pathogens, such as equine herpesvirus, may result in more severe reproductive outcomes, increased neonatal mortality, and greater production losses [9, 10]. Beyond immediate reproductive failure, outbreaks impose considerable financial burdens through laboratory diagnostics, quarantine measures, movement restrictions, replacement of breeding stock, and disruption of long-term genetic improvement programs. In high-value breeding operations, cumulative economic losses may reach hundreds of thousands of US dollars due to foal mortality, reduced reproductive efficiency, prolonged breeding intervals, and disruptions to breeding schedules.

The increasing prevalence of multidrug-resistant *Salmonella* isolates in equids, together with reports of environmental contamination in horse and donkey production systems, has intensified concerns about the broader implications of *S. *Abortusequi infection within the One Health framework. The circulation of antimicrobial-resistant strains not only complicates therapeutic management in equine practice but also raises concerns regarding the dissemination of antimicrobial resistance (AMR) determinants among animals, humans, and the environment. Consequently, strengthening epidemiological surveillance, antimicrobial stewardship, and integrated disease control strategies has become increasingly important for protecting both animal and public health.

Despite considerable advances in the understanding of *S. *Abortusequi over recent decades, the available evidence remains fragmented across epidemiological investigations, diagnostic studies, molecular characterization, AMR surveillance, and prevention strategies. Most published reports focus on isolated outbreaks or regional investigations, with limited integration of global epidemiological trends, advances in rapid diagnostics, genomic epidemiology, vaccine development, bacteriophage therapy, and emerging control strategies. Furthermore, variations in diagnostic methodologies, geographical reporting bias, inconsistent surveillance systems, and the limited field validation of novel diagnostic and preventive approaches hinder comprehensive interpretation of the available evidence. To date, no recent review has comprehensively synthesized these rapidly evolving aspects within a unified One Health perspective while critically identifying current knowledge gaps and future research priorities.

Therefore, this review aims to comprehensively synthesize and critically evaluate the current evidence regarding the global epidemiology, pathogenesis, diagnosis, prevention, AMR, molecular epidemiology, and emerging control strategies for *S. *Abortusequi-associated abortions in horses and other equids. Particular emphasis is placed on recent advances in serological and molecular diagnostics, whole-genome sequencing (WGS), AMR surveillance, vaccine development, bacteriophage therapy, and integrated One Health approaches for disease surveillance and control. By consolidating contemporary evidence published between 2000 and 2025, this review seeks to provide veterinarians, researchers, diagnosticians, and animal health authorities with an updated scientific resource to support improved disease surveillance, early diagnosis, evidence-based prevention, and sustainable long-term control of *S. *Abortusequi infection in equine populations.

## REVIEW METHODOLOGY

### Literature sources

The literature search was conducted using the electronic databases PubMed, ScienceDirect, BASE, and Google Scholar to identify peer-reviewed studies relevant to *S. *Abortusequi-associated abortions in horses. Additional publications were identified through manual screening of the reference lists of eligible articles and review papers. The review primarily included studies published between 2000 and 2025 to provide an up-to-date assessment of the global epidemiology, diagnostic advances, prevention strategies, AMR, molecular epidemiology, and emerging control measures for *S. *Abortusequi. Earlier landmark publications were included when they provided important historical background or methodological information. Only articles published in English or Russian were considered for inclusion.

### Literature search strategy

A comprehensive literature search was performed using combinations of keywords related to *S. *Abortusequi and equine salmonellosis. The search terms included "*Salmonella *Abortusequi", "Salmonella abortion in mares," "equine salmonellosis," "horse abortion," "donkey abortion," "*Salmonella enterica*," "epidemiology," "distribution," "diagnostics," "enzyme-linked immunosorbent assay (ELISA)," "polymerase chain reaction (PCR)," "immunochromatographic assay (ICA)," "vaccination," "bacteriophage therapy," "whole-genome sequencing," "antimicrobial resistance," "molecular epidemiology," "prevention," and "equine infectious diseases." These terms were searched individually and in various combinations using Boolean operators (AND and OR) to maximize retrieval of relevant publications across the selected databases.

### Inclusion and exclusion criteria

Studies were considered eligible if they investigated *S. *Abortusequi or other *Salmonella* serovars associated with equine abortion or salmonellosis and reported information related to epidemiology, diagnostic methods, prevention, molecular characterization, AMR, or disease control. Studies involving horses, donkeys, mules, or other equids were included. Only full-text articles published in English or Russian between 2000 and 2025 were eligible for inclusion.

Studies were excluded if full-text articles were unavailable, methodological descriptions or analytical data were insufficient, the reported findings were incomplete or unclear, or the publications were written in languages other than English or Russian.

### Data extraction and qualitative synthesis

Initially, the titles and abstracts of all retrieved publications were independently screened to determine their relevance. Subsequently, the full texts of potentially eligible articles were critically reviewed, and relevant information was extracted and categorized according to predefined thematic areas. The extracted data were qualitatively synthesized to summarize global trends in the epidemiology and geographical distribution of *S. *Abortusequi, evaluate advances in diagnostic technologies, compare preventive and vaccination strategies, assess AMR patterns, summarize developments in bacteriophage therapy, and examine the contribution of molecular epidemiological approaches, particularly WGS, to understanding pathogen diversity, evolution, and transmission dynamics.

### Scope of the review

This review provides a comprehensive synthesis of current knowledge regarding the epidemiology, diagnosis, prevention, molecular epidemiology, AMR, and emerging control strategies for *S. *Abortusequi-associated abortions in horses and other equids. Particular emphasis is placed on contemporary diagnostic approaches, including bacteriological culture, ELISA, PCR-based methods, ICA, and point-of-care molecular diagnostic techniques. In addition, recent advances in vaccination strategies, antimicrobial stewardship, bacteriophage therapy, and WGS are critically evaluated for their applications in phylogenetic analysis, molecular surveillance, outbreak investigation, and disease control. Finally, this review discusses the current limitations of existing diagnostic and preventive approaches and highlights future opportunities to develop rapid, highly sensitive, field-applicable, and sustainable strategies for the surveillance, prevention, and long-term control of equine salmonellosis.

## DISTRIBUTION AND GLOBAL EPIDEMIOLOGY

Published evidence demonstrates that *S. *Abortusequi infection is widely distributed among equine populations across multiple continents, although its reported prevalence varies considerably according to geographical region, surveillance intensity, and diagnostic methodology. Several studies from China have documented the circulation of *S. *Abortusequi in horses. For example, Mai *et al.* [11] detected specific antibodies against *S. *Abortusequi in 20.91% of equids using ELISA, indicating widespread exposure within the investigated populations.

Although reports of salmonellosis-associated equine abortion have been relatively infrequent since the 1980s, leading to an underestimation of its veterinary importance, China has experienced a noticeable reemergence of the disease over the past decade, with an increasing number of reported cases [12]. Gao *et al.* [13] reported a high prevalence of *S. *Abortusequi in horses from four provinces in northwestern China within the context of the One Health framework, detecting seroprevalence and molecular prevalence rates of 35.03% and 23.03%, respectively. In Japan, *Salmonella* infection has also been documented in draft horses [14]. High serum agglutination titers (RA ≥1:2560) were detected, and *S. *Abortusequi was isolated from a thrombus within the cranial mesenteric artery during necropsy. These findings suggest that aneurysms of the cranial mesenteric artery may serve as potential sites for bacterial persistence in infected horses [15]. Furthermore, pulsed-field gel electrophoresis (PFGE) and fluorescent amplified-fragment length polymorphism analyses demonstrated close genetic relatedness among *S. *Abortusequi isolates from Japan and other countries, suggesting a common evolutionary origin of these strains [16].

Although *S. *Abortusequi is traditionally recognized as the principal cause of abortion in horses, recent studies have demonstrated its pathogenic potential in donkeys. In China, *S. *Abortusequi was identified in nine aborted donkey fetuses and confirmed as the etiological agent responsible for the abortions [17]. Although the mechanisms underlying abortion in donkeys remain poorly understood, these findings indicate that *S. *Abortusequi can infect both horses and donkeys. Another Chinese study confirmed *Salmonella*-associated abortions in donkeys and successfully established a mouse abortion model to investigate disease pathogenesis. Histopathological and immunofluorescence analyses demonstrated similar pathological changes in pregnant mice and donkeys following infection [18].

In Pakistan, *Salmonella* isolates were recovered from horses, donkeys, and mules [19]. In India, 45 *S. *Abortusequi isolates obtained from horses were characterized for AMR profiles [20]. Babu *et al.* [21] reported a salmonellosis prevalence of 14.2% in horses and identified 11 different *Salmonella* serovars, of which 75.7% exhibited multidrug resistance. Another investigation reported *Salmonella* isolation rates of 6.5%, 3.8%, and 23.3% from horses, donkeys, and mules, respectively. PCR detected *Salmonella* DNA in 10.8% of horses, 4.9% of donkeys, and 65.1% of mules, confirming the higher diagnostic sensitivity of molecular methods compared with conventional culture [22]. Similarly, another study demonstrated that PCR was the most sensitive and specific method for detecting *Salmonella* in equine feces, while tetrathionate broth was identified as the most suitable selective enrichment medium for bacterial isolation [23].

In Russia, *Salmonella*-associated abortions have been reported primarily in the Republic of Sakha (Yakutia), Novosibirsk Region, Krasnoyarsk Krai, and Altai Krai, where herd horse breeding is extensively practiced. Farms affected by the disease recorded foal crops ranging from only 30% to 55% [24]. Disease transmission has been associated with high animal density, harsh climatic conditions, and reduced natural resistance in pregnant mares during the winter-spring season [25]. Environmental persistence also contributes to disease maintenance. Neustroev and Ordakhov reported that *S. *Abortusequi remained viable beneath snow for up to 120 days at ambient temperatures ranging from −14°C to −19°C, highlighting the remarkable environmental resilience of the pathogen [26, 27].

Evidence from Europe indicates that *S. *Abortusequi has not been eliminated despite previous assumptions. In Croatia, abortion occurred in 21 of 26 pregnant mares within a herd of 38 horses between the fifth and tenth months of gestation without preceding clinical signs. Histopathological and bacteriological examinations of aborted fetuses identified *S. *Abortusequi as the sole causative pathogen, confirming its continued circulation in Europe [28].

Similarly, Marenzoni *et al.* [29] investigated 67 equine abortion cases, 22 stillbirths, and 14 neonatal deaths from 31 farms comprising purebred horses, working horses, and Shetland ponies. *Salmonella* spp. were isolated from two aborted fetuses and identified as *S. enterica* subsp. *enterica* serovars Bareilly and Abortusequi. In Italy, a severe outbreak occurred among Murgese horses in 2018, during which 10 of 34 foals were stillborn and seven additional foals died within 10 days after developing severe clinical disease. Sixteen *S. *Abortusequi isolates were recovered from the parenchymal tissues of two deceased foals [30]. Bolzoni *et al.* [31] subsequently investigated horses entering the slaughter process in northern Italy and detected *Salmonella* in cecal contents from 3 of 146 horses (2.0%), demonstrating that clinically healthy horses may serve as asymptomatic carriers. Additional reports have documented *Salmonella* isolation from horses in Germany, the Netherlands, and the United Kingdom [32–34].

In South America, *S. *Abortusequi continues to represent an important reproductive pathogen. In Argentina, an outbreak occurred at a polo pony breeding farm where 29 abortions were recorded among 120 unvaccinated pregnant mares. High antibody titers were detected serologically, and pure cultures of *S. *Abortusequi (4,12:-:e,n,x) were isolated from aborted fetuses. Similar outbreaks were subsequently reported on neighboring farms [35]. Researchers from the University of Buenos Aires also documented major outbreaks of equine paratyphoid characterized predominantly by abortions in pregnant mares [36]. Comprehensive pathological, molecular, and bacteriological investigations confirmed *S. *Abortusequi as the causative agent [37]. PFGE analysis of 27 Argentine isolates collected between 2011 and 2016 demonstrated substantial genetic diversity, suggesting multiple infection sources or ongoing bacterial evolution [38]. In addition, multidrug-resistant (MDR) *S. *Typhimurium has been isolated from equine clinical cases in Argentina [39].

Confirmed cases of equine salmonellosis have also been reported in Brazil [40]. In Chile, 22 *Salmonella* isolates were recovered from environmental surfaces within an equine veterinary hospital, including stalls, operating rooms, and water troughs, while one isolate was recovered directly from a horse. Serotyping identified 18 isolates as *S. *Typhimurium and three as *S. *Infantis, emphasizing the importance of environmental contamination in disease transmission [41].

In North America, *Salmonella* was isolated from fecal samples collected from 13 of 230 hospitalized horses in the United States [42]. A large epidemiological investigation involving 972 horse farms across 28 states reported *Salmonella* fecal shedding in 0.8% of horses and contamination of 0.4% of grain and concentrate feed samples [43]. In Atlantic Canada, retrospective surveillance identified *S. enterica* subsp. *enterica* serovar Oranienburg in 4.6% (n = 154) of equine samples collected between 2012 and 2021 [44]. Earlier reports from Ontario documented 17 cases of DT104-associated equine salmonellosis between 1997 and 2000, highlighting concerns regarding multidrug resistance and zoonotic transmission [45].

In Australia, MDR *Salmonella *Heidelberg isolates were recovered from horses admitted to a veterinary clinic in Victoria [46]. Additional studies documented *Salmonella* isolation from foals [47], adult horses infected with *S. *Typhimurium [48], and abortion outbreaks among Thoroughbred mares during 2021–2022 [49]. More recently, surveillance across 11 Thoroughbred stud farms in New South Wales and Victoria during 2023–2024 detected *Salmonella* in 5 of 1,476 fecal samples collected from 1,330 horses, indicating relatively low but persistent pathogen circulation [50].

In Africa, *Salmonella* infection has been documented in horses admitted to veterinary clinics in South Africa [51]. Furthermore, the Onderstepoort Veterinary Institute reported 1,229 *Salmonella* isolations, including 116 (9.4%) originating from horses, confirming the continued occurrence of equine salmonellosis in the region [52].

Kazakhstan represents one of the countries where *S. *Abortusequi remains particularly important because horse breeding constitutes a major component of the livestock industry. Sultanov *et al.* [53] reported abortion rates ranging from 6% to 30% among mares in southern Kazakhstan, with approximately 50% of abortions attributed to *Salmonella* infection. Shokanov *et al.* [54] confirmed by PCR that *S. *Abortusequi is the predominant bacterial cause of equine abortion and recommended ELISA-based surveillance to identify latent carriers and reduce economic losses. Additional investigations confirmed pathogen circulation in both herd-managed horses and privately owned animals in northern Kazakhstan [55]. Issabekov *et al.* [56] reported that 14.3% of 217 equine abortion cases investigated between 2021 and 2024 were caused by *S. *Abortusequi. Similarly, Bakishev *et al.* [57] detected PCR positivity rates ranging from 25.0% to 33.3% among horses from nine regions of Kazakhstan, while ELISA identified antibodies against *S. *Abortusequi in 10.6% of the sampled animals.

Collectively, the available literature demonstrates that *S. *Abortusequi-associated abortion remains an important infectious disease of equids worldwide. Between 2000 and 2025, outbreaks have been documented across Asia, Europe, South America, North America, Africa, and Australia. Notable events include a high mortality outbreak among Italian foals in 2018, an abortion outbreak followed by emergency vaccination in Italy in 2022, abortion outbreaks in jennies in China during 2019, the isolation of *S. *Abortusequi from aborted fetuses and vaginal swabs in Xinjiang, China, in 2023, and recurrent outbreaks documented in Kazakhstan during 2015, 2023, and 2025. These findings emphasize the continued global circulation of *S. *Abortusequi and reinforce the need for strengthened surveillance, standardized diagnostic strategies, and coordinated international control programs.

**DIAGNOSTIC APPROACHES FOR S. ABORTUSEQUI INFECTION**

Accurate diagnosis of equine salmonellosis remains challenging because of the diverse clinical manifestations of the disease, the occurrence of subclinical infections, and intermittent bacterial shedding. These factors hinder early case detection, facilitate environmental contamination, and complicate disease control programs [58]. Although abortion and characteristic pathological lesions in aborted fetuses strongly suggest *S. *Abortusequi infection, definitive diagnosis requires isolation and identification of the causative organism [59].

According to the guidelines of the World Organization for Animal Health (WOAH; formerly OIE), bacteriological examination remains the reference standard for diagnosing *Salmonella*-associated abortion. Conventional diagnosis involves bacterial isolation, biochemical characterization, and serotyping according to the Kaufmann–White–Le Minor scheme [60]. However, bacteriological culture has several limitations, including relatively low sensitivity, dependence on sample quality, and prolonged turnaround time (≥3 days) [61]. Consequently, WOAH recommends complementing conventional bacteriology with modern diagnostic methods, including ELISA and PCR, to improve diagnostic sensitivity and facilitate rapid confirmation of infection.

### Serological diagnosis

Several ELISA formats have been developed for detecting antibodies against *S. *Abortusequi in equine serum. One of the earliest approaches utilized antigen-capture ELISA (AC-ELISA), which reduced diagnostic turnaround time by approximately 3 days compared with conventional bacteriological culture [62]. Nevertheless, a negative AC-ELISA result does not exclude *Salmonella* infection, and bacterial culture remains necessary for definitive confirmation. Subsequently, an indirect ELISA (iELISA) targeting the lipopolysaccharide antigen of *S. *Abortusequi demonstrated improved performance compared with the conventional tube agglutination test (TAT) for detecting serum antibodies [63].

Singh *et al.* [64] reported a seroprevalence of 26.2% among breeding mares in northern India using ELISA, demonstrating the usefulness of serological surveillance in endemic regions. Likewise, Mai *et al.* [11] investigated the prevalence of *S. *Abortusequi infection in horses from Xinjiang, China, between 2020 and 2023. Among 971 serum samples analyzed by ELISA, antibodies were detected in 20.91% of horses. Seropositivity was higher in mares than in stallions (22.45% vs. 14.05%) and was considerably greater in purebred horses than in indigenous breeds (43.22% vs. 11.24%). Furthermore, young and older horses exhibited higher seroprevalence (70.00% and 41.86%, respectively) than adult animals (19.79%).

Guo *et al.* [65, 66] demonstrated that the flagellin gene *fljB* could differentiate *S. *Abortusequi from other *Salmonella* serovars. Based on this finding, the authors developed a competitive ELISA (cELISA) utilizing recombinant flagellin antigen for detecting antibodies in both horses and donkeys. Evaluation using 660 positive and 515 negative serum samples showed that cELISA outperformed TAT in terms of diagnostic efficiency, speed, and ease of interpretation, making it suitable for large-scale serological surveillance.

In Kazakhstan, iELISA has been successfully applied for the serological diagnosis of equine salmonellosis. Antibodies against *S. *Abortusequi were detected in 20%–43% of horses from unvaccinated herds, supporting its utility for evaluating the epizootic status of equine populations [67]. Another study demonstrated that an outer membrane protein antigen of *S. *Abortusequi exhibited greater diagnostic specificity than lipopolysaccharide-based assays [68]. Collectively, these findings indicate that ELISA substantially improves the speed and efficiency of serological diagnosis; however, its diagnostic performance depends on the availability of highly specific antigens and high-quality antibodies. Furthermore, recombinant outer membrane protein X of *S. enterica* has also demonstrated considerable promise as a serological antigen for diagnosing *Salmonella*-associated abortion in mares [69].

### Molecular diagnostic methods

Conventional PCR, multiplex PCR, and real-time PCR have become indispensable tools for diagnosing *Salmonella* infections because of their high sensitivity, specificity, and rapid turnaround time [70–72]. PCR assays developed for equine fecal samples detected *Salmonella* DNA in 40% of specimens, whereas conventional bacteriological culture identified only 2% as positive, highlighting the superior analytical sensitivity of molecular diagnostics [73]. Similarly, real-time PCR demonstrated a relative sensitivity of 100% and specificity of 98.2% compared with bacteriological culture, making it particularly valuable during outbreak investigations and herd surveillance [74].

To facilitate rapid diagnosis of abortions associated with *S. *Abortusequi, a real-time PCR assay targeting the *fljB* gene was developed and evaluated using 540 clinical samples collected in China between 2018 and 2021. The assay demonstrated 100% agreement with conventional bacteriological diagnosis, confirming its suitability for rapid clinical diagnosis [75]. Another investigation evaluated quantitative real-time PCR by incorporating cycle threshold (Ct) values into the interpretation of results. The authors demonstrated that Ct values improve diagnostic interpretation and provide additional clinical information beyond simple pathogen detection [76].

More recently, point-of-care PCR (POC-PCR) systems have emerged as promising diagnostic tools for veterinary practice. One POC-PCR platform demonstrated concordance rates of 88.1% for fecal samples and 97.0% for environmental samples compared with quantitative PCR, supporting its application for rapid on-site diagnosis in equine hospitals [77]. Similarly, another POC-PCR assay evaluated in the United States showed excellent agreement with laboratory-based quantitative PCR and was recommended for environmental surveillance in high-risk areas of equine veterinary facilities [78].

### Rapid point-of-care diagnostic technologies

Although ELISA and PCR substantially improve diagnostic accuracy, both require laboratory infrastructure, specialized equipment, and trained personnel. Consequently, rapid field-deployable diagnostic methods have attracted increasing attention. Immunochromatographic assay (ICA) represents one of the most practical alternatives because it enables rapid pathogen detection directly at horse housing facilities without laboratory support. An ICA developed to detect *Salmonella* in equine fecal samples reliably identified *S. enterica* after approximately 18 h of enrichment. However, additional validation using naturally infected animals is required before routine clinical implementation [79].

The RapidChek® SELECT™ *Salmonella* system further reduces diagnostic turnaround time by combining double enrichment with ICA, providing results within 22–44 h compared with approximately 5 days required for the standard culture protocol. Comparative studies demonstrated excellent sensitivity, specificity, and diagnostic accuracy relative to matrix-assisted laser desorption/ionization time-of-flight mass spectrometry [80].

Phage display technology has also facilitated the development of highly specific antibodies for ICA without requiring animal immunization. Using phage-derived antibody fragments, investigators developed an ICA that specifically detects *Salmonella *Enteritidis, providing an additional platform for rapid pathogen identification [81].

To further improve analytical sensitivity, investigators combined recombinase polymerase amplification with a streptavidin-biotin-based ICA. This platform transmitted results directly to smartphone-based software and achieved a detection limit of 19.41 colony-forming units (CFU)/mL for *Salmonella *Enteritidis, demonstrating the potential of digital-assisted rapid diagnostics [82]. Another innovative multiplex ICA incorporating magnetic quantum dots simultaneously detected *Pseudomonas aeruginosa* and *S. *Typhimurium, achieving pathogen capture efficiencies exceeding 80%, detection limits below 30 cells/mL, assay completion within 35 min, and excellent reproducibility [83].

### Emerging molecular diagnostic technologies

Recent advances in molecular diagnostics have led to the integration of loop-mediated isothermal amplification (LAMP) with clustered regularly interspaced short palindromic repeats (CRISPR)/Cas12b technology for *Salmonella* detection. This single-tube LAMP–CRISPR/Cas12b platform performs amplification and detection within less than 1 h without requiring sophisticated laboratory equipment. The assay demonstrated a detection limit of only 12.5 DNA copies per reaction while maintaining excellent analytical specificity, highlighting its considerable potential as a rapid, highly sensitive, and field-deployable diagnostic platform for *Salmonella* surveillance and outbreak management [84].

## PLACENTAL TROPISM AND MECHANISMS OF ABORTION

The placenta forms a critical physical and immunological barrier between the maternal and fetal compartments, thereby limiting the transmission of pathogens during pregnancy [85]. However, bacteria of the genus *Salmonella* have evolved mechanisms that enable immune evasion, survival within host cells, and transplacental dissemination, potentially resulting in placental injury, fetal compromise, and maternal complications [86]. Nevertheless, the precise mechanisms through which *S.* Abortusequi infection causes reproductive failure and pregnancy-associated complications remain incompletely understood.

Evidence indicates that *S.* Abortusequi infection may contribute to impaired conception, abortion, early intrauterine fetal death, fetal maceration, and fetal resorption [87]. The potential role of *S.* Abortusequi in infertility was also demonstrated experimentally. Subclinical infection of guinea pigs with an isogenic wild-type strain of *S.* Abortusequi adversely affected fertility, whereas infection with the *aroA*, *htrA*, and *aroA–htrA* mutant strains had minimal effects on conception rates. These findings suggest that the products encoded by *aroA* and *htrA* may contribute to reproductive pathogenicity and persistence within the host [88].

Betancourt *et al.* [89] demonstrated that *S.* Abortusequi infection may have prolonged consequences extending beyond placental development and fetal growth to neonatal viability and early postnatal development. Singh *et al.* [90] further established that the host specificity of *S.* Abortusequi is associated, at least partly, with its ability to survive and multiply within equine macrophages. Intracellular persistence within macrophages may facilitate systemic dissemination, immune evasion, and colonization of reproductive tissues. However, further studies are required to characterize the molecular pathways involved in placental invasion, inflammatory injury, fetal infection, and reproductive loss in the natural equine host.

## PREVENTION THROUGH VACCINATION

Vaccination is an important preventive measure to control the spread of *Salmonella* infection in equine populations, particularly in endemic regions and high-value breeding farms. In an Italian study, pregnant mares were immunized with a commercial *S.* Abortusequi vaccine, which contributed to effective outbreak control [30]. In addition, a vaccine formulated with Montanide™ Seppic IMS1313 adjuvant demonstrated high efficacy against equine salmonellosis without detectable adverse effects [91]. However, complete product information, including the manufacturer, city, and country, should be provided at its first mention in accordance with journal requirements.

**Three principal vaccine categories have been investigated for the control of salmonellosis:** inactivated bacterins, subunit vaccines, and live attenuated vaccines. A major limitation of several available formulations is their restricted cross-protection against heterologous *Salmonella* serovars. Therefore, genetically modified strains and conserved antigen-based platforms may be required to develop vaccines that provide broader and more durable protection [92, 93].

In India, an *aroA* mutant strain, B-26, of *S.* Abortusequi was evaluated as a potential vaccine candidate [94]. Female guinea pigs immunized orally or intramuscularly demonstrated 100% protection against salmonellosis, abortion, and mortality for 135–165 days after challenge with the wild-type E-156 strain. Although these findings demonstrate strong protective efficacy in an experimental model, additional safety, immunogenicity, and challenge studies are required in horses before this candidate can be recommended for field application.

Another Indian study investigated three deletion mutants, Δ*aroA*, Δ*htrA*, and Δ*aroA*Δ*htrA*, as vaccine candidates against abortions in mares and foal mortality caused by *S.* Abortusequi. The double-deletion mutant Δ*aroA*Δ*htrA* produced the highest protective efficacy in guinea pigs and showed particular promise as a live attenuated vaccine candidate against equine salmonellosis [95]. Nevertheless, the duration of immunity, protection throughout gestation, potential shedding of the vaccine strain, and genetic stability of the deletion mutants must be assessed in the target species.

In Russia, a vaccine based on the *S.* Abortusequi BN-12 strain was developed and shown to be effective in preventing *Salmonella*-associated abortion in horses [96]. Similarly, a live attenuated vaccine derived from the *S.* Abortusequi E-841 strain has been successfully used in Kazakhstan to prevent abortion in pregnant mares [97].

Pregnancy loss among purebred mares represents a major economic concern for stud farms. In Australia, *Salmonella* spp. were identified as an important cause of abortion among purebred mares during 2021–2022, reinforcing the potential value of vaccination in populations at increased risk of infection [98]. However, vaccination should be integrated with surveillance, isolation of affected animals, safe disposal of aborted materials, environmental disinfection, movement control, and monitoring of bacterial shedding rather than being used as a stand-alone intervention.

## EMERGING BACTERIOPHAGE-BASED CONTROL STRATEGIES

Bacteriophages have emerged as promising biological tools for controlling bacterial pathogens, including non-typhoidal *S. enterica*. The increasing prevalence of antimicrobial-resistant strains poses a serious challenge to animal and public health and has stimulated interest in phage therapy, as bacteriophages can selectively target bacterial pathogens without extensively disrupting the beneficial host microbiota. Phage-derived lytic proteins, including endolysins, have also demonstrated activity against several bacterial pathogens, including antimicrobial-resistant strains [99].

The immunogenic efficacy of a phage lysate was evaluated in a guinea pig model, in which both humoral and cellular immune responses were detected. This approach represents a novel method of bacterial inactivation and may support the development of safe immunoprophylactic preparations against equine salmonellosis [100]. However, the protective efficacy, optimal dose, safety, and duration of immunity associated with such preparations require validation in horses.

Wang *et al.* [101] evaluated the prophylactic and therapeutic efficacy of bacteriophage PIZ SAE-01E2 against *S.* Abortusequi in a mouse model. The phage remained stable across broad temperature and pH ranges and lysed 25 of 28 tested *Salmonella* strains belonging to serogroups O:4 and O:9. A single intraperitoneal administration of PIZ SAE-01E2, either before or after infection, protected all pregnant mice. These findings indicate that this phage may have potential for preventing abortion caused by *S.* Abortusequi *in vivo*. Nevertheless, its efficacy and safety in pregnant mares remain to be established.

Bacteriophages have also been investigated as biological control agents for foodborne pathogens. Phage vB_SalP_LDDK01, isolated from a donkey farm, did not contain detectable virulence or AMR genes. The phage inhibited the growth of *S.* Abortusequi and reduced viable bacterial counts, suggesting that it may be useful for limiting *Salmonella* contamination in donkey meat [102].

Jaglan *et al.* [103] isolated the lytic bacteriophage phiSalP219 from pond water for activity against MDR *S. enterica* serovars Paratyphi. The phage lysed 28 of 30 tested *S. enterica* strains, showed no identified microbiological safety concerns, and disrupted *Salmonella* biofilms formed on borosilicate glass surfaces. In addition, polymer-based encapsulation systems with improved acid resistance have been developed to enhance the stability and oral delivery of anti-*Salmonella* bacteriophages [104]. Such delivery technologies may help protect phages from gastric degradation and improve their bioavailability at the intended site of action.

Although *S.* Abortusequi is classically associated with abortion in mares, recent evidence has confirmed its capacity to induce abortion in donkeys. The virulent bacteriophage vB_SabS_Sds2, designated Sds2, was isolated from donkey feces using *S.* Abortusequi as the bacterial host. Sds2 exhibited strong bactericidal activity against *S.* Abortusequi over a broad range of multiplicities of infection and prevented abortion when administered *in vivo* to experimentally infected mice [105]. These findings further support the potential application of phages against reproductive salmonellosis in different equid species.

Despite their promising prophylactic and therapeutic potential, several limitations must be addressed before bacteriophage-based interventions can be implemented in equine practice. These include regulatory barriers associated with approval, production standardization, quality control, storage stability, formulation, dosing, and clinical validation of phage preparations. The safety of phage therapy in pregnant mares also requires careful evaluation, particularly regarding potential effects on placental function, fetal development, reproductive outcomes, immune responses, and the maternal microbiome. In addition, bacteria may develop resistance to individual phages, potentially reducing therapeutic efficacy. This limitation may be mitigated by developing multivalent phage cocktails, routinely monitoring bacterial susceptibility, and periodically updating phage formulations. Large-scale controlled studies in naturally infected horses are therefore required before bacteriophage therapy can be incorporated into routine prevention or outbreak control programs.

## WGS AND MOLECULAR EPIDEMIOLOGY

WGS and phylogenomic analysis have substantially advanced the understanding of *S.* Abortusequi by enabling comprehensive characterization of its genome and elucidation of evolutionary relationships among circulating strains. These approaches provide high-resolution information regarding virulence determinants, AMR genes, mobile genetic elements, phylogenetic relationships, and pathogen evolution, thereby improving epidemiological investigations and outbreak tracing.

Bustos *et al.* investigated the genetic diversity of *S.* Abortusequi isolates recovered in Argentina between 2011 and 2016 using WGS and molecular typing approaches [38]. Four virulence profiles and nine PFGE pulsotypes were identified. Most isolates shared a common virulence profile and carried all investigated virulence-associated genes except *gipA* and *sopE1*, which are associated with intestinal virulence. These findings suggest that although *S.* Abortusequi populations are genetically related, considerable genomic diversity exists among circulating field strains.

Researchers in China successfully isolated *Salmonella* strains from vaginal swabs collected from aborted mares and subsequently performed WGS. Genomic analysis confirmed that strain XJ2032 belonged to *S.* Abortusequi and harbored numerous virulence-associated genes and AMR determinants. The study further demonstrated that genomic islands and bacteriophages contribute substantially to the pathogenicity, adaptation, and genetic diversity of *S.* Abortusequi [106, 107].

Another Chinese investigation characterized the *S.* Abortusequi strain ST251 (XJP1), which was isolated from a donkey. Comparative genomic analysis demonstrated that XJP1 was closely related to *S. enterica* subsp. *enterica* strains isolated from humans, horses, donkeys, and poultry and shared genetic characteristics with multiple *Salmonella* serovars. The isolate carried an IncFIB/IncFII incompatibility plasmid containing the *spvBC* operon, together with several important virulence-associated genes, including *rck*, type III secretion system (TTSS)-associated genes, *pefABCD*, *fdeC*, and the acid resistance regulator *gadX*. Collectively, these genes may contribute to bacterial survival, host adaptation, and pathogenicity [108].

An Indian study performed WGS and comparative genomic analysis of *S.* Abortusequi isolated from a donkey. Core genome multilocus sequence typing (cgMLST) demonstrated that the investigated isolate formed a distinct phylogenetic clade compared with previously reported *S.* Abortusequi strains, suggesting ongoing genomic evolution within this serovar [109].

In Kazakhstan, Musayeva *et al.* [110] performed molecular characterization of the vaccine strain *S.* Abortusequi E-841 and compared it with virulent field isolates. Sequencing of the *rpoB* and *rpsL* genes confirmed the genetic stability of the vaccine strain and facilitated the identification of mutations associated with its biological characteristics and safety.

Another study conducted the first whole-genome characterization and phylogenetic analysis of three *S.* Abortusequi isolates recovered from aborted equine specimens in Kazakhstan. All isolates exhibited high genomic similarity and were classified as *S.* Abortusequi with the antigenic profile 4:a:e,n,x. Comparative genomic analysis identified twelve *Salmonella* pathogenicity islands and three prophages, including ST64B, which was present in all isolates. These findings demonstrate the considerable genetic conservation and virulence potential of *S.* Abortusequi strains circulating in Kazakhstan [111].

Although not specifically focused on equine isolates, a Korean study investigated *S. enterica* strains recovered from poultry farms using WGS. The analysis revealed substantial genomic diversity among three *Salmonella* serovars and demonstrated the utility of *in silico* genomic approaches for accurate serovar prediction and identification of genetic determinants associated with AMR. These findings further illustrate the broader application of WGS in *Salmonella* surveillance and molecular epidemiology [112].

## AMR AND GENOMIC SURVEILLANCE

The increasing prevalence of antimicrobial-resistant *Salmonella* strains has become a major concern for both veterinary medicine and public health. WGS has emerged as a valuable tool for simultaneously identifying AMR genes, virulence determinants, plasmids, and phylogenetic relationships, thereby supporting surveillance and antimicrobial stewardship programs.

An American study analyzed 200 *S. enterica* serovar Infantis isolates obtained from animal sources using WGS. Resistance to at least one antimicrobial agent was detected in 33.5% of isolates, whereas 19.5% were classified as MDR. Importantly, this investigation confirmed the emergence of the MDR ESI clone across multiple animal species and documented the first occurrence of a pESI-like plasmid in equine isolates from the United States [113].

Another study investigating horses, donkeys, and mules isolated *Salmonella* from 42 (6.5%), 7 (3.8%), and 10 (23.3%) samples, respectively. Remarkably, 63 of the 65 recovered isolates exhibited MDR phenotypes, emphasizing the increasing dissemination of antimicrobial-resistant *Salmonella* among equids [114].

The Animal Health Diagnostic Center and the New York State Veterinary Diagnostic Laboratory isolated group C2 *Salmonella* strains from horses on four farms in New Jersey and Pennsylvania. All isolates demonstrated identical AMR profiles characterized by resistance to multiple commonly used antimicrobial agents, suggesting clonal dissemination among geographically separated farms [115].

Phenotypic and genotypic characterization of AMR was also performed using *S. enterica* isolates recovered from 2,182 horses in Kentucky. Eleven serotypes were identified, of which 11.4% exhibited MDR phenotypes [116]. Similarly, genomic investigations of *Salmonella* isolates recovered from horses admitted to a veterinary hospital in the southern United States between 2007 and 2015 identified phylogenetic relationships among common serotypes, temporal changes in serotype distribution, and diverse mechanisms of AMR. These findings provide valuable information for developing targeted disease control strategies and evidence-based antimicrobial stewardship policies in equine medicine [117].

A Polish study investigated *Salmonella* strains isolated from reptiles using PFGE and virulence gene profiling. The authors demonstrated that exotic reptiles frequently act as asymptomatic carriers of *Salmonella* serovars distinct from those commonly associated with human salmonellosis, including *S.* Enteritidis and *S.* Typhimurium. The international trade of exotic reptiles may therefore facilitate the introduction of previously unrecognized *Salmonella* serovars into European countries [118]. Although this study did not involve horses, it illustrates the broader epidemiological importance of cross-species pathogen dissemination and emphasizes the relevance of One Health surveillance.

To facilitate genomic surveillance, investigators from the United States and the United Kingdom established a comprehensive genomic database containing approximately 10,000 *S. enterica* isolates collected between 1891 and 2010 from 73 countries. The database includes isolates originating from humans, domestic animals, reptiles, environmental water sources, and other reservoirs, substantially expanding the publicly available genomic diversity of *S. enterica* and providing an invaluable resource for comparative genomic analyses, phylogenetic investigations, and global surveillance programs [119].

Complementing genomic surveillance, Switzerland implemented a voluntary veterinary epidemiological surveillance system for equine infectious diseases that are not subject to mandatory notification. Veterinary reports submitted between November 2013 and April 2019 were evaluated to assess reporting quality, participant engagement, population coverage, geographical representativeness, and reporting timeliness. This surveillance model demonstrates how integrated epidemiological monitoring can complement molecular surveillance to facilitate the early detection and control of emerging infectious diseases in equine populations [120].

**GLOBAL DISTRIBUTION OF S. ABORTUSEQUI INFECTION**

Our review demonstrates that *S.* Abortusequi infection has been reported in equine populations across numerous countries worldwide. The circulation of *Salmonella* infection has been documented in Asia, including China, Japan, India, Kazakhstan, Pakistan, South Korea, and Thailand; Europe, including Russia, Croatia, Italy, Belgium, Germany, the Netherlands, the Czech Republic, and the United Kingdom; North America; South America; Australia; and Africa. Collectively, these reports indicate that *S.* Abortusequi remains an important cause of reproductive disease in equids with a broad geographical distribution. The published literature describing the global occurrence of *Salmonella*-associated abortions in horses, donkeys, and mules is summarized in Table 1.

**Table 1:** Global distribution of published reports describing Salmonella-associated abortions and Salmonella infections in horses, donkeys, and mules (2000–2025).

**Continent**	**Country**	**Articles reporting infections in horses, n**	**Articles reporting infections in donkeys, n**	**Articles reporting infections in mules, n**	**References**
**Europe**	Belgium	1	–	–	[77]
	Italy	4	–	–	[29–31, 89]
	Germany	1	–	–	[32]
	Netherlands	1	–	–	[33]
	Russia	5	–	–	[24–27, 94]
	Croatia	1	–	–	[28]
	Czech Republic	1	–	–	[32]
	United Kingdom	1	–	–	[34]
**Asia**	India	17	2	1	[20–23, 64, 85, 86, 88, 90–93, 98, 101, 102, 106, 111]
	China	17	5	2	[11–13, 17, 18, 65, 66, 71, 75, 82–84, 99, 100, 103–105]
	Kazakhstan	11	–	–	[53–57, 67–69, 95, 107, 108]
	Japan	3	–	–	[14–16]
	South Korea	1	–	–	[109]
	Pakistan	1	1	1	[19]
	Thailand	1	–	–	[81]
**Africa**	South Africa	2	–	–	[51, 52]
**North America**	United States	13	–	–	[42, 43, 74, 76, 78–80, 97, 110, 112–114, 116]
	Canada	4	–	–	[44, 45, 63, 72]
**South America**	Argentina	6	–	–	[35–39, 87]
	Brazil	1	–	–	[40]
	Chile	1	–	–	[41]
**Australia**	Australia	7	–	–	[46–50, 73, 96]

**Figure 1:**
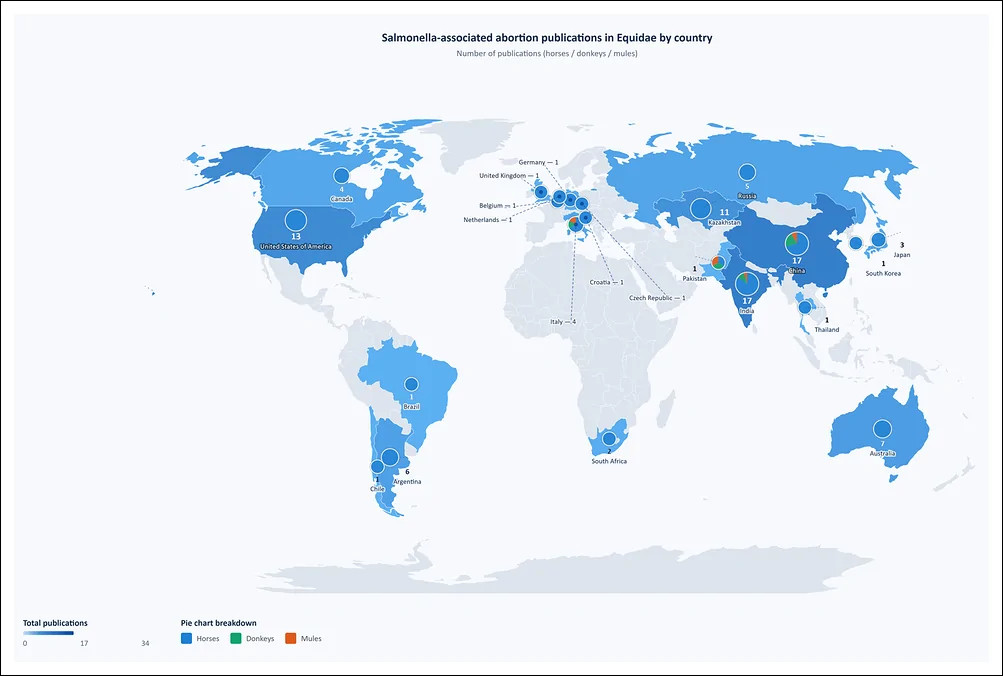
Global distribution and publication intensity of studies on *Salmonella* Abortusequi-associated abortions in equids published between 2000 and 2025. The size of each circle represents the number of publications retrieved from the literature, whereas the pie charts indicate the proportion of studies involving horses, donkeys, and mules.

As summarized in Table 1, published studies have documented either *Salmonella*-associated abortions or the isolation of *Salmonella* strains from horses, donkeys, and mules in at least 22 countries worldwide. These reports collectively demonstrate the widespread geographical distribution of *S.* Abortusequi and related *Salmonella* infections in equids. The global distribution of the published studies is illustrated in Figure 1.

## COMPARATIVE EVALUATION OF DIAGNOSTIC METHODS

Early and accurate diagnosis represents the cornerstone of effective surveillance, outbreak control, and eventual eradication of equine salmonellosis. According to WOAH recommendations, bacteriological examination remains the reference method for confirming *Salmonella*-associated abortion; however, conventional culture is limited by relatively low analytical sensitivity and prolonged turnaround time. Consequently, ELISA- and PCR-based diagnostic methods have become increasingly important for serological surveillance and rapid pathogen detection in cases of *Salmonella*-associated abortion [58–78].

In addition to laboratory-based molecular and serological assays, ICAs have emerged as valuable rapid diagnostic tools because they facilitate the detection of either specific antibodies or *Salmonella* antigens directly from clinical or environmental samples collected at horse housing facilities. Their portability, simplicity, and rapid turnaround make them particularly useful for field investigations and preliminary outbreak screening [79–83].

More recently, the integration of LAMP with CRISPR/Cas12b technology has provided a highly promising molecular platform for *Salmonella* detection. This single-tube assay combines rapid amplification with highly specific nucleic acid detection, enabling sensitive diagnosis without sophisticated laboratory infrastructure. Such technologies have considerable potential to strengthen epidemiological surveillance, improve outbreak investigations, and support public health monitoring within a One Health framework [84].

The principal diagnostic methods currently available for detecting *S.* *Abortusequi* and equine salmonellosis, together with their diagnostic performance, advantages, and limitations, are summarized in Table 2.

## PLACENTAL PATHOGENESIS AND REPRODUCTIVE CONSEQUENCES

The placenta serves as a critical physical and immunological barrier that protects the developing fetus from invading pathogens during pregnancy in both animals and humans [85]. However, *Salmonella* species have evolved sophisticated mechanisms that enable them to evade host immune responses, survive within phagocytic cells, and cross the placental barrier, resulting in fetal infection, placental damage, and adverse pregnancy outcomes [86]. Experimental studies using animal models have demonstrated that *S.* Abortusequi infection is associated with impaired conception, abortion, early intrauterine fetal death, fetal resorption, and infertility [87, 88]. Furthermore, the host specificity of *S.* Abortusequi appears to be closely associated with its capacity to survive and multiply within equine macrophages, facilitating intracellular persistence and systemic dissemination [89, 90]. These findings suggest that placental colonization, intracellular survival, and immune evasion are central mechanisms underlying reproductive failure caused by *S.* Abortusequi.

**Table 2:** Comparison of diagnostic methods for detecting Salmonella Abortusequi and equine salmonellosis.

**Diagnostic method**	**Target detected**	**Sample type**	**Diagnostic performance**	**Time to result**	**Advantages**	**Limitations**	**References**
Bacteriological culture, biochemical identification, and serotyping	Viable *Salmonella*	Aborted fetuses, fetal membranes, feces, environmental samples	Reference method; high specificity	≥3–5 days	Gold standard; enables bacterial isolation, antimicrobial susceptibility testing, and serotyping	Time-consuming; lower sensitivity; influenced by sample quality	[60, 61]
AC-ELISA	*Salmonella* antigens	Feces	Sensitivity 69%; specificity 97%	Several hours	Rapid screening	Negative results do not exclude infection	[62]
Indirect ELISA (LPS-based)	Anti-*S.* Abortusequi antibodies	Serum	Not reported	Several hours	High throughput; suitable for herd surveillance	Detects exposure rather than active infection; possible cross-reactivity	[63]
Competitive ELISA (*fljB* antigen)	Anti-*S.* Abortusequi antibodies	Serum	AUC 0.9941 (95% CI: 0.9898–0.9984); sensitivity 98.03%; specificity 99.81%	Several hours	Suitable for horses and donkeys; simple interpretation	Requires validated antigen and laboratory infrastructure	[65]
Recombinant *fljB*-based iELISA	Anti-*S.* Abortusequi *fljB* antibodies	Serum	Sensitivity 95.6%; specificity 99.5%; AUC 0.992	3–4 h	Highly specific recombinant antigen; suitable for large-scale surveillance; reduced cross-reactivity	Detects previous exposure; requires recombinant antigen production and laboratory facilities	[66]
Recombinant OmpX-based indirect ELISA	Anti-recombinant OmpX antibodies	Serum	High specificity and diagnostic accuracy; evaluated using sera from aborted and contact mares	Several hours	Improved specificity; suitable for serological surveillance	Requires recombinant protein production; additional validation required	[69]
Hexa-plex PCR	Multiple *Salmonella* genetic targets	Clinical and food samples	Simultaneous detection of *Salmonella* spp. and five major serovars	4–8 h	Simultaneous detection and serotyping	Does not specifically identify *S.* Abortusequi; requires PCR laboratory	[71]
Conventional PCR	*Salmonella* DNA	Feces, clinical samples	Detected DNA in 40% of samples versus 2% by culture	4–8 h	Rapid and highly sensitive	Requires molecular laboratory facilities	[73]
Real-time PCR	*Salmonella* DNA	Enriched fecal samples	Sensitivity 100%; specificity 98.2% compared with culture	Same day after enrichment	Rapid; highly sensitive; suitable for outbreak investigations	Requires enrichment and specialized equipment; detects DNA rather than bacterial viability	[74]
Real-time PCR targeting *fljB*	*S.* Abortusequi-specific DNA	Clinical samples	100% agreement with bacteriological culture (540 samples)	2–4 h	Highly specific; useful during outbreaks	Requires molecular laboratory infrastructure	[75]
Quantitative real-time PCR (Ct interpretation)	*Salmonella* DNA	Equine feces	Ct cutoff of 30.4 associated with severe SIRS (AUC = 0.84)	2–4 h	Provides prognostic as well as diagnostic information	Ct values require assay standardization	[76]
Point-of-care PCR (targets *invA* and *ttrC*)	*Salmonella* DNA	Pooled environmental samples	100% agreement with qPCR	~24 h	Rapid on-site surveillance; reduced testing cost	Requires enrichment and dedicated POC platform	[77]
Point-of-care PCR	*Salmonella* DNA	Equine feces and environmental samples	Agreement with qPCR: 88.1% (feces) and 97.0% (environment)	Same day after enrichment	Near-patient testing; suitable for outbreak management	Requires enrichment; detects DNA rather than viable bacteria	[78]
Immunochromatographic assay	*Salmonella* antigens	Feces, food, environmental samples	Reliable detection following enrichment	Approximately 18 h	Rapid and simple field test	Requires enrichment; limited validation using naturally infected horses	[79]
RapidChek® SELECT™ *Salmonella*	*Salmonella* antigen	Environmental samples	High sensitivity and specificity	22–44 h	Faster than conventional culture; suitable for environmental surveillance	Requires enrichment and culture confirmation	[80]
Colorimetric lateral flow assay using phage-derived antibodies	Live *S.* Enteritidis	Bacterial cultures, food samples	Highly specific; no cross-reactivity observed	~15 min	Very rapid; simple visual interpretation	Developed for *S.* Enteritidis rather than *S.* Abortusequi	[81]
RPA-ICA with streptavidin-biotin amplification	*S.* Enteritidis DNA	Clinical and food samples	Detection limit: 19.41 CFU/mL	<1 h	Highly sensitive; smartphone-assisted interpretation	Not validated for equine clinical samples	[82]
Magnetic quantum dot ICA	*S.* Typhimurium and *Pseudomonas aeruginosa*	Clinical and food samples	Capture efficiency >80%; detection limit <30 cells/mL	<35 min	Multiplex; highly sensitive	Experimental platform; not specific for *S.* Abortusequi	[83]
LAMP–CRISPR/Cas12b	*Salmonella* DNA	Clinical and food samples	Detection limit: 12.5 copies/reaction	<1 h	Single-tube assay; rapid; no specialized equipment required	Experimental; limited field validation	[84]

AC-ELISA: Antigen-capture enzyme-linked immunosorbent assay; AUC: Area under the receiver operating characteristic curve; CFU: Colony-forming units; CI: Confidence interval; Ct: Cycle threshold; ELISA: Enzyme-linked immunosorbent assay; ICA: Immunochromatographic assay; iELISA: Indirect enzyme-linked immunosorbent assay; LAMP: Loop-mediated isothermal amplification; LPS: Lipopolysaccharide; OmpX: Outer membrane protein X; PCR: Polymerase chain reaction; POC-PCR: Point-of-care polymerase chain reaction; qPCR: Quantitative polymerase chain reaction; RPA: Recombinase polymerase amplification; SIRS: Systemic inflammatory response syndrome; TAT: Tube agglutination test; WOAH: World Organization for Animal Health.

## VACCINATION STRATEGIES FOR PREVENTION

Vaccination remains one of the most effective strategies for preventing *Salmonella*-associated abortion, improving reproductive performance, and reducing economic losses in horse breeding. During an outbreak in Italy, pregnant mares received two booster doses of a commercial *S.* Abortusequi vaccine at 21-day intervals, followed by two additional booster vaccinations using an autogenous *S.* Abortusequi bacterin. This integrated vaccination strategy successfully controlled disease transmission and terminated the outbreak [30].

A vaccine formulated with the adjuvant Montanide™ Seppic IMS1313 was subsequently registered in Italy for the prevention of *S.* Abortusequi infection in combination with targeted antimicrobial therapy. The vaccine produced high antibody titers without detectable adverse effects and may reduce the risk of future outbreaks in endemic populations [91].

Several vaccine candidates have also been developed in India for the prevention of *Salmonella*-associated abortion [92–95]. Among these, attenuated mutants carrying deletions in *aroA* and *htrA* have demonstrated promising protective efficacy in guinea pig models. The double-deletion mutant (Δ*aroA*Δ*htrA*) provided the highest level of protection while exhibiting an acceptable safety profile, suggesting that rationally attenuated live vaccines may represent a promising direction for future vaccine development [94, 95].

In the Russian Federation, an inactivated vaccine based on the *S.* Abortusequi BN-12 strain was developed for preventing *Salmonella*-associated abortion in horses. Field evaluations demonstrated effective protection against infectious abortion, with an estimated economic return of 14.1 rubles for every ruble invested in vaccination [96]. Similarly, Kazakhstan successfully introduced a live attenuated vaccine based on the *S.* Abortusequi B-0147 strain for preventing abortion in pregnant mares [97]. In Australia, the importance and feasibility of vaccinating pregnant Thoroughbred mares against salmonellosis have also been recognized because of the substantial reproductive and economic losses associated with disease outbreaks [98].

The principal vaccines and vaccine candidates currently investigated for the prevention of equine salmonellosis are summarized in Table 3.

**Table 3:** Comparison of vaccines and vaccine candidates developed for the prevention of equine salmonellosis.

**Vaccine/** **candidate**	**Commercial name**	**Developer/** **manufacturer**	**Vaccine type**	**Vaccination schedule/route**	**Duration of immunity**	**Protective efficacy**	**Safety**	**DIVA capability**	**References**
Autogenous *Salmonella* Abortusequi vaccine with Montanide™ Seppic IMS1313	Experimental candidate	Autogenous formulation; Montanide™ IMS1313 adjuvant (Seppic)	Inactivated autogenous adjuvanted vaccine	Emergency vaccination during an outbreak; second dose administered after reaction to the aluminum hydroxide formulation	Approximately 12 months	Successfully halted outbreak progression and induced high protective antibody titers	No adverse effects reported with the IMS1313 formulation	Not reported	[91]
B-26 Δ*aroA* mutant of *S.* Abortusequi	Experimental candidate	Indian Veterinary Research Institute (IVRI), India	Live attenuated mutant	Oral and intramuscular immunization in a guinea pig model	>5.5 months	Provided 100% protection against abortion and mortality following challenge	Demonstrated acceptable safety in the experimental model	Potential marker vaccine; not fully established	[94]
Δ*aroA*, Δ*htrA*, and Δ*aroA*Δ*htrA* mutants	Experimental candidate	IVRI, India	Live attenuated deletion mutants	Oral, mucosal, and subcutaneous administration evaluated in guinea pigs	>4.5 months	All mutants protected against abortifacient challenge; Δ*aroA*Δ*htrA* showed the highest protective efficacy	Δ*htrA* caused mortality following intraperitoneal inoculation, whereas Δ*aroA*Δ*htrA* did not induce abortion in pregnant guinea pigs	Δ*aroA*Δ*htrA* considered to have DIVA potential	[95]
*S.* Abortusequi BN-12 strain with *Bacillus subtilis* TNP-3 filtrate	Experimental candidate	Yakut Research Institute of Agriculture and the All-Russia Research Institute of Experimental Veterinary Medicine	Inactivated vaccine	Single intramuscular injection (2 cm³) into the upper third of the neck	At least 7 months	Effective in preventing *Salmonella*-associated abortion; favorable economic return reported	Not reported	Not reported	[96]
Live attenuated *S.* Abortusequi B-0147 strain	Dry live vaccine against *Salmonella* abortion in mares	Kazakh Scientific Research Veterinary Institute and Research Institute for Biological Safety Problems	Live attenuated dry vaccine	Two subcutaneous injections administered 10 days apart during the 4th–7th months of gestation	Protective immunity develops within 2 weeks and persists for approximately 12 months	Reported to be highly immunogenic and protective against *Salmonella*-associated abortion in pregnant mares	Not reported	Not reported	[97]

DIVA: Differentiating infected from vaccinated animals

As demonstrated by field experience, vaccination represents a reliable and cost-effective strategy for protecting equine reproductive health and maintaining foal production. The economic benefits of vaccination substantially outweigh the financial losses associated with abortion, neonatal mortality, veterinary treatment, biosecurity measures, and disinfection procedures. Nevertheless, no currently available vaccine provides broad cross-protection against all clinically relevant *Salmonella* serovars. Developing vaccines capable of inducing durable, cross-serovar immunity therefore remains one of the major challenges in controlling equine salmonellosis.

## EMERGING BACTERIOPHAGE-BASED CONTROL STRATEGIES

Bacteriophage therapy has emerged as one of the most promising alternatives to conventional antimicrobial therapy because of the remarkable host specificity of bacteriophages and their ability to selectively eliminate bacterial pathogens without substantially disrupting the normal microbiota. In addition to therapeutic applications, novel bacteriophage-derived immunoprophylactic approaches have also been proposed for the prevention of equine salmonellosis [99].

Among the investigated phages, PIZ SAE-01E2 demonstrated particularly promising results in a mouse model of *S.* Abortusequi infection. The bacteriophage provided complete protection against abortion when administered either prophylactically or therapeutically, indicating its potential utility for preventing *S.* Abortusequi-associated abortion *in vivo* [101]. Another bacteriophage, vB_SalP_LDDK01, effectively inhibited the growth of *S.* Abortusequi in contaminated donkey meat and may therefore contribute to improving food safety [102].

Jaglan *et al.* [103] isolated the lytic bacteriophage phiSalP219, which exhibited activity against MDR *S. enterica* Paratyphi serovars and lysed numerous *S. enterica* isolates while effectively disrupting *Salmonella* biofilms formed on borosilicate glass surfaces. Furthermore, polymer-based encapsulation technologies have been developed to improve the stability and oral delivery of anti-*Salmonella* bacteriophages, thereby enhancing their therapeutic potential [104].

To combat *Salmonella*-associated abortion in donkeys, the bacteriophage vB_SabS_Sds2 (Sds2) was isolated and shown to possess strong bactericidal activity against *S.* Abortusequi. Administration of this phage prevented abortion in experimentally infected mice, suggesting considerable promise for future therapeutic applications [105].

The currently available bacteriophages and phage-based strategies investigated for controlling *Salmonella* infections relevant to equine salmonellosis are summarized in Table 4.

Bacteriophage therapy represents one of the most promising emerging strategies for controlling *Salmonella*-associated abortion in horses, particularly considering the increasing prevalence of AMR and the limited cross-protective efficacy of existing vaccines. Among the investigated candidates, bacteriophage 4FS1 appears especially promising because it combines broad lytic activity, excellent environmental stability, the absence of detectable virulence and AMR genes, and production of the depolymerase Dpo36, which has demonstrated antibiofilm activity and protective effects against *S.* Abortusequi *in vivo* [106].

Although several bacteriophages, particularly PIZ SAE-01E2, Sds2, and 4FS1, have demonstrated encouraging efficacy, most investigations have been limited to laboratory experiments or mouse models. Consequently, additional studies using naturally infected horses are required to establish safety in pregnant mares, optimal dosing regimens, pharmacokinetic properties, routes of administration, long-term efficacy, and the potential emergence of phage-resistant bacterial variants before routine veterinary application can be recommended.

**Table 4:** Bacteriophages and phage-based approaches investigated for the control of Salmonella infections relevant to equine salmonellosis.

**Bacteriophage/** **phage-based approach**	**Target organism**	**Host range**	**Stability characteristics**	***In vivo*** ** efficacy**	**Administration/** **application**	**References**
PIZ SAE-2 (phage lysate)	*Salmonella* Abortusequi	Based on phage-mediated bacterial lysis products rather than direct phage therapy	Not reported	Induced both humoral and cellular immune responses and protected guinea pigs against challenge with virulent *S.* Abortusequi	Intraperitoneal administration; evaluated as an experimental immunoprophylactic approach	[100]
PIZ SAE-01E2	*S.* Abortusequi	Lysed most tested *Salmonella* strains belonging to serogroups O:4 and O:9 (25/28 isolates)	Stable over a broad range of temperatures and pH values	Completely protected pregnant mice when administered before or after *S.* Abortusequi infection	Intraperitoneal administration in mice; proposed for both prophylactic and therapeutic applications	[101]
vB_SalP_LDDK01	*S.* Abortusequi	Broad lytic activity against *S.* Abortusequi isolates	Stable after incubation at 40°C, 50°C, and 60°C for 1 h; maintained activity at pH 4–12	Reduced viable bacterial counts and inhibited bacterial growth in contaminated donkey meat	Food biocontrol application for reducing *Salmonella* contamination in donkey meat	[102]
phiSalP219	Multidrug resistant *S. enterica* serovars (including Paratyphi)	Lysed 28 of 30 tested *S. enterica* isolates	Demonstrated microbiological safety and disrupted *Salmonella* biofilms	No animal efficacy studies reported	Potential application for food safety, environmental decontamination, and biofilm control	[103]
vB_SabS_Sds2 (Sds2)	*S.* Abortusequi	Strong bactericidal activity over a wide range of multiplicities of infection	Stable lytic activity against *S.* Abortusequi	Prevented abortion in experimentally infected mice	*In vivo* administration in a mouse abortion model	[105]
4FS1 and depolymerase Dpo36	*S.* Abortusequi S1 and other *Salmonella* spp.	4FS1 lysed 36 of 60 tested *Salmonella* strains	Stable at 37–50°C; active at pH 5–10; retained activity after 1 h of ultraviolet exposure; remained stable in 2.5%–15% NaCl for 4 h	Dpo36 combined with donkey serum improved survival and reduced bacterial loads in mice challenged with *S.* Abortusequi	Proposed for therapy of intestinal *Salmonella* infection in donkeys; authors suggested administration through salt blocks; Dpo36 evaluated *in vitro* and *in vivo*	[106]

## WGS AND AMR

WGS and phylogenomic analyses have become indispensable tools for investigating the molecular epidemiology of *S.* Abortusequi. These technologies facilitate accurate pathogen identification, monitoring of genomic evolution, outbreak source tracing, identification of virulence determinants, and characterization of AMR genes. In Argentina, genotyping of *S.* Abortusequi isolates identified four virulence profiles and nine pulsotypes, although most isolates shared a common virulence profile [38]. In China, WGS of an isolate recovered from an aborted mare identified numerous virulence-associated and AMR genes [107]. Similarly, the XJP1 strain isolated from a donkey showed close phylogenetic relationships with *S. enterica* subsp. *enterica* strains recovered from humans, horses, donkeys, and poultry, indicating possible evolutionary relationships among isolates from different hosts [108]. In India, cgMLST demonstrated that an investigated *S.* Abortusequi isolate formed a distinct phylogenetic clade compared with previously described strains, suggesting continued genomic diversification [109].

In Kazakhstan, whole-genome characterization of *S.* Abortusequi isolates demonstrated high genomic similarity while identifying twelve *Salmonella* pathogenicity islands and three prophages, highlighting the conserved virulence architecture of circulating strains [110, 111].

The emergence of MDR *Salmonella* strains remains a growing concern in veterinary medicine and One Health. In the United States, WGS of *S. enterica* serovar Infantis isolates demonstrated resistance to at least one antimicrobial agent in 33.5% of isolates, whereas 19.5% were classified as MDR [112]. Similarly, Indian investigators reported that nearly all *Salmonella* isolates recovered from horses, donkeys, and mules exhibited MDR phenotypes [113]. Group C2 *Salmonella* isolates recovered from four horse farms in New Jersey and Pennsylvania displayed highly similar AMR profiles, suggesting clonal dissemination among geographically separated premises [114]. In Kentucky, 11 different *Salmonella* serotypes were identified among equine isolates, with 11.4% classified as MDR [115]. Additional genomic investigations involving horses admitted to veterinary hospitals in the southern United States have further characterized the phenotypic and genotypic mechanisms underlying AMR. Collectively, these findings provide valuable information for designing targeted disease control strategies and developing evidence-based antimicrobial stewardship policies in equine veterinary medicine [116].

## KNOWLEDGE GAPS, LIMITATIONS, AND FUTURE PERSPECTIVES

Despite considerable advances in understanding *S.* Abortusequi, important knowledge gaps remain regarding its epidemiology, molecular evolution, pathogenesis, diagnostics, AMR, and prevention. The true global burden of *S.* Abortusequi infection is likely underestimated because of limited surveillance systems, underreporting of abortion cases, inadequate laboratory capacity, and the absence of standardized diagnostic protocols in many horse breeding regions. Although modern diagnostic techniques, including ELISA, PCR, ICA, and WGS, have substantially improved pathogen detection and molecular characterization, many of these technologies still require large-scale validation under diverse field conditions before they can be routinely implemented. Furthermore, the number of publicly available genomic sequences of *S.* Abortusequi remains relatively limited, restricting a comprehensive understanding of pathogen evolution, host adaptation, virulence mechanisms, transmission dynamics, and the emergence and dissemination of AMR determinants.

Future research should prioritize the establishment of coordinated international surveillance networks to improve the detection and reporting of *S.* Abortusequi infections in horses and other equids. Standardization of diagnostic protocols and validation of rapid, highly sensitive, and field-deployable diagnostic platforms should also be considered research priorities to facilitate early disease detection and outbreak control. In addition, expanded application of WGS and comparative genomic analyses involving isolates from different geographical regions is required to improve understanding of pathogen evolution, molecular epidemiology, virulence-associated genes, and AMR mechanisms. Further investigations should evaluate the long-term efficacy, duration of immunity, and cross-protective capacity of existing and novel vaccine candidates under field conditions. Similarly, large-scale studies are needed to assess the safety, pharmacokinetics, therapeutic efficacy, and practical implementation of bacteriophage-based interventions in naturally infected horses. Integrated disease control strategies that combine molecular epidemiological surveillance, vaccination, biosecurity measures, antimicrobial stewardship, and innovative biological control approaches are expected to provide more sustainable and effective prevention and control of *S.* Abortusequi-associated abortion in equine populations.

Several limitations should be considered when interpreting the findings of this review. First, the available literature is geographically heterogeneous, with most published studies originating from China, India, Russia, Kazakhstan, and a limited number of European countries, whereas information from many important horse breeding regions remains scarce or unavailable. Second, considerable heterogeneity exists among the included studies with respect to study design, sample size, animal populations, diagnostic methodologies, outcome measures, and reporting formats. Consequently, direct comparison of prevalence estimates, abortion rates, diagnostic performance, and intervention outcomes across different studies was not feasible, precluding formal quantitative meta-analysis. Third, evidence regarding the effectiveness of vaccines, bacteriophage therapy, and several molecular diagnostic and genomic approaches remains limited because many investigations have been performed under experimental conditions or involved relatively small numbers of animals. Furthermore, this review included only publications available in English and Russian, which may have introduced a language bias and led to the exclusion of relevant studies published in other languages. The limited availability of regional surveillance reports, conference proceedings, governmental veterinary records, and other non-indexed literature may also have contributed to publication bias and an incomplete representation of the global epidemiology of *S.* Abortusequi. Despite these limitations, this review provides one of the most comprehensive and up-to-date syntheses of the available evidence on the epidemiology, diagnosis, prevention, molecular epidemiology, AMR, vaccination, and emerging control strategies for *S.* Abortusequi-associated abortion in equids. It also identifies important research priorities that may guide future investigations and support the development of evidence-based surveillance and disease control programs.

## CONCLUSION

*S.* Abortusequi remains one of the most important bacterial causes of abortion in horses, resulting in substantial reproductive losses and significant economic consequences for the equine industry worldwide. This review demonstrates that *S.* Abortusequi infection has been reported across Asia, Europe, North and South America, Africa, and Australia, confirming its broad geographical distribution and continued importance as an emerging reproductive pathogen. Advances in serological and molecular diagnostic methods, including ELISA, PCR, ICA, and WGS, have considerably improved the rapid detection, epidemiological investigation, and molecular characterization of *S.* Abortusequi. Furthermore, WGS has enhanced understanding of pathogen evolution, virulence-associated genes, and AMR, while novel bacteriophage-based approaches have emerged as promising alternatives for controlling infections caused by MDR strains.

Despite these advances, important challenges remain. Conventional bacteriological diagnosis is labor-intensive and time-consuming, available vaccines provide limited cross-serovar protection, and most bacteriophage-based therapies have been evaluated only under experimental conditions. In addition, the limited availability of global surveillance data and genomic information continues to restrict understanding of the epidemiology, transmission dynamics, and evolution of *S.* Abortusequi. Consequently, integrated surveillance systems and harmonized diagnostic approaches are needed to improve early detection and outbreak management.

A major strength of this review is its comprehensive synthesis of current evidence spanning epidemiology, pathogenesis, diagnosis, vaccination, AMR, WGS, and emerging control strategies within a One Health framework. However, the interpretation of the available evidence is constrained by geographical disparities in published studies, heterogeneity in study methodologies and diagnostic approaches, and the limited number of field evaluations for vaccines, bacteriophage therapy, and advanced molecular technologies.

Future research should prioritize international epidemiological surveillance, large-scale genomic investigations, validation of rapid, field-deployable diagnostic platforms, long-term evaluation of vaccine efficacy, and well-designed clinical studies to assess the safety and effectiveness of bacteriophage therapy in naturally infected horses. Integrating molecular epidemiology, biosecurity measures, antimicrobial stewardship, vaccination, and innovative biological control strategies will be essential for reducing reproductive losses and limiting the spread of antimicrobial-resistant *Salmonella* strains. Overall, continued multidisciplinary research and coordinated international disease control programs are fundamental for improving the prevention, surveillance, and sustainable control of *S.* Abortusequi-associated abortion in equine populations.

## DATA AVAILABILITY

The supplementary data can be made available from the corresponding author upon a request.

## AUTHORS’ CONTRIBUTIONS

TB: Conceptualization, comprehensive literature search, data collection, investigation, and manuscript writing. ZB: Data curation, compilation of tables, preparation of the global distribution map, and manuscript review. SA: Data synthesis, formal analysis, interpretation of the literature, drafting of the manuscript, and manuscript review. SB: Conceptualization, supervision, critical review, editing, proofreading, and validation of the manuscript. All authors have read and approved the final version of the manuscript.
